# Structural Violence and Clinical Medicine: Free Infant Formula for HIV-Exposed Infants

**DOI:** 10.1371/journal.pmed.0040087

**Published:** 2007-02-27

**Authors:** Ted Greiner, Christophe Grundmann, Katherine Krasovec, Christian Pitter, Catherine Wilfert

We wholeheartedly agree with Paul Farmer and colleagues [1] that it is vitally important to examine social, as well as molecular, causes of disease. Unless we carefully consider the full range of factors that underlie a given problem, we may produce “solutions” with unintended and deleterious consequences. In this light we express our concern about the infant feeding approach advocated in their article to reduce mother-to-child transmission of HIV in Rwanda.

While exclusive replacement feeding reduces the risk of transmission between HIV-positive mothers and their infants, it does not adequately address the specters of infection and undernutrition that accompany avoidance of breast-feeding. We are convinced—by data from regions that are similar to Rwanda and even from African countries with higher standards of living—that replacement feeding from birth is a dangerous and inappropriate approach for HIV-affected families in countries like Rwanda.

In addition, avoiding breast-feeding from birth can be exceedingly risky, particularly in the same regions where the risk of mother-to-child transmission of HIV is highest. While Partners in Health (PIH) offers high-quality health-care support and financial assistance to reduce the risks associated with breast-feeding avoidance in two districts in Rwanda, it is impossible to eliminate those risks. Researchers have found that children in Ghana, Peru, and India who are not breast-fed between the ages of six weeks and six months have a ten-fold higher risk of death [2]. A multi-country analysis by the World Health Organization (WHO) showed that infants who were born to mothers with little education and were not breast-fed had a five-fold increased risk of death from six to 11 months of age. Since about 5% of breast-fed Rwandan babies already die in the first six months of life and another 3.5% from six to12 months [3], it is essential that PIH substantiate the mortality, nutrition, and morbidity outcomes resulting from their approach before promoting it more widely.

Given that breast-feeding avoidance increases the risk of death from other causes, even as it decreases the risk of HIV transmission, is there a net gain? The concept of “HIV-free survival” combines the likelihood of surviving with the likelihood of not becoming HIV infected, allowing a more comprehensive assessment of the risks and benefits of infant feeding. In Botswana [4] and the Ivory Coast [5], rates of HIV-free survival were no better among formula-fed infants than among infants breast-fed for three to six months. At this year's WHO Consultation on HIV and Infant Feeding in Geneva, reports showed high death rates in ongoing trials in Kenya, Uganda, and Malawi associated with breast-feeding cessation at three to six months. These results were despite earlier assumptions that breast-feeding cessation at this age might be safe, while avoiding most of the HIV transmission associated with prolonged breast-feeding [6]. Since these carefully controlled studies represent best-case scenarios for replacement feeding, most actual program settings will favor breast-feeding (actually, disfavor replacement feeding).

The risk of mother-to-child HIV transmission in the first six months in a country like Rwanda, where 81% of women are still exclusively breast-feeding at four to six months [3], is relatively low—probably approximately 0.3% per month [7]. It may be even lower in districts like those in which PIH works, where eligible HIV-positive mothers begin receiving highly active antiretroviral therapy during pregnancy, because the majority of postnatal HIV transmission is from mothers with low CD4+ cell counts [8].

Scientific evidence amply demonstrates the significant risks that accompany replacement feeding and the safety and effectiveness of exclusive breast-feeding for the first six months, and continued breast-feeding thereafter as appropriate and safe. Around the world, researchers, programmers, and policy makers are becoming increasingly convinced that the infant feeding counseling component of prevention of mother-to-child transmission of HIV programs must focus on optimizing HIV-free survival rates, not simply on HIV transmission. Accomplishing this means taking full account of all factors, both social and molecular, that are at work in a particular context, and tailoring responses to meet them.
